# A Preliminary Study on the Pattern, the Physiological Bases and the Molecular Mechanism of the Adductor Muscle Scar Pigmentation in Pacific Oyster *Crassostrea gigas*

**DOI:** 10.3389/fphys.2017.00699

**Published:** 2017-09-12

**Authors:** Wenchao Yu, Cheng He, Zhongqiang Cai, Fei Xu, Lei Wei, Jun Chen, Qiuyun Jiang, Na Wei, Zhuang Li, Wen Guo, Xiaotong Wang

**Affiliations:** ^1^School of Agriculture, Ludong University Yantai, China; ^2^Changdao Enhancement and Experiment Station, Chinese Academy of Fishery Sciences Changdao, China; ^3^Institute of Oceanology, Chinese Academy of Sciences Qingdao, China; ^4^Research Center of Marine Molluscs, Marine Biology Institute of Shandong Province Qingdao, China

**Keywords:** Pacific oyster, pigmentation, adductor muscle scar, outer surface of shell, melanin, dried soft-body weight

## Abstract

The melanin pigmentation of the adductor muscle scar and the outer surface of the shell are among attractive features and their pigmentation patterns and mechanism still remains unknown in the Pacific oyster *Crassostrea gigas*. To study these pigmentation patterns, the colors of the adductor muscle scar vs. the outer surface of the shell on the same side were compared. No relevance was found between the colors of the adductor muscle scars and the corresponding outer surface of the shells, suggesting that their pigmentation processes were independent. Interestingly, a relationship between the color of the adductor muscle scars and the dried soft-body weight of Pacific oysters was found, which could be explained by the high hydroxyl free radical scavenging capacity of the muscle attached to the black adductor muscle scar. After the transcriptomes of pigmented and unpigmented adductor muscles and mantles were studied by RNAseq and compared, it was found that the retinol metabolism pathway were likely to be involved in melanin deposition on the adductor muscle scar and the outer surface of the shell, and that the different members of the tyrosinase or Cytochrome P450 gene families could play a role in the independent pigmentation of different organs.

## Introduction

As we all know, shell color is one of most attractive features of mollusks. Current research into the pigmentation of mollusks is mainly focused on carotenoids in the shell and soft body (Li et al., 2010; Zheng et al., 2010, 2012; Maoka, 2011; Maoka et al., 2014; Liu et al., 2015; Williams, 2017), which have roles in quenching singlet oxygen species, eliminating free radicals, acting as antioxidants, and supporting the immune system (Rao and Rao, 2007; Maiani et al., 2009). Melanin has a similar biological function as carotenoids (Kollias et al., 1991; Sharma et al., 2002), and it has been confirmed that the black pigment in the soft body, outer surface of the shell, and adductor muscle scar of oysters is melanin (Hao et al., 2015; Yu et al., 2015; Williams, 2017).

About the genetic mechanism of oyster shell color, there are different opinions. In previous cases, researchers viewed Pacific oyster shell pigmentation as a continuously distributed, quantitative trait under polygenic control (Brake et al., 2004; Batista et al., 2008), but in recent cases, shell coloration was determined to be controlled by a small quantity of major genes or be under relatively high genetic control with the moderate-to-high narrow-sense heritability value (Sanford et al., 2009; Ge et al., 2015; Wan et al., 2017). Comparative transcriptome analysis of the left shell color variants (white, golden, black, and partially pigmented) of Pacific oyster has also been carried out, and it was found that one tyrosinase gene may be correlated with golden coloration of left shell but no tyrosinase gene with black coloration (Feng et al., 2015). The adductor muscle scar is part of the shell wall secreted at the site of attachment of the adductor muscles functioning to close/open the shells (Lee et al., 2011). The diversity of adductor muscle scar color was also observed in *Crassostrea angulata* or *Crassostrea gigas* (Batista et al., 2008; Higuera-Ruiz and Elorza, 2011).

However, the relationship between the adductor muscle scar color and the shell color remains unclear. Thus, in the current study, the colors of the adductor muscle scars and the outer surfaces of the shell were compared, the relationship between the pigmentation pattern and the soft-body dry weight was determined, and the genes involved in the melanin pigmentation on the adductor muscle scar and the outer surface of the shell were analyzed in the Pacific oyster.

## Materials and methods

### Experimental animals

The oysters used in pigmentation pattern analysis were acquired from three farms located in Yantai, Rushan, and Penglai in Shandong Province, China, and produced in hatchery. Three hundred oysters were collected from each location, and their average shell heights in three locations were 9.4 ± 0.5, 8.4 ± 0.6, and 8.4 ± 0.4 cm, respectively.

The oysters, in the experiment of antioxidant physiology indexes detection, were obtained from market (Zhifu, Yantai, China). They were dissected and the color of the adductor muscle scars were observed. The muscles attached to white adductor scar (called “white muscle” for short) and the muscles attached to black adductor scar (called “black muscle” for short) were sampled and stored at −80°C.

**Figure 1:**
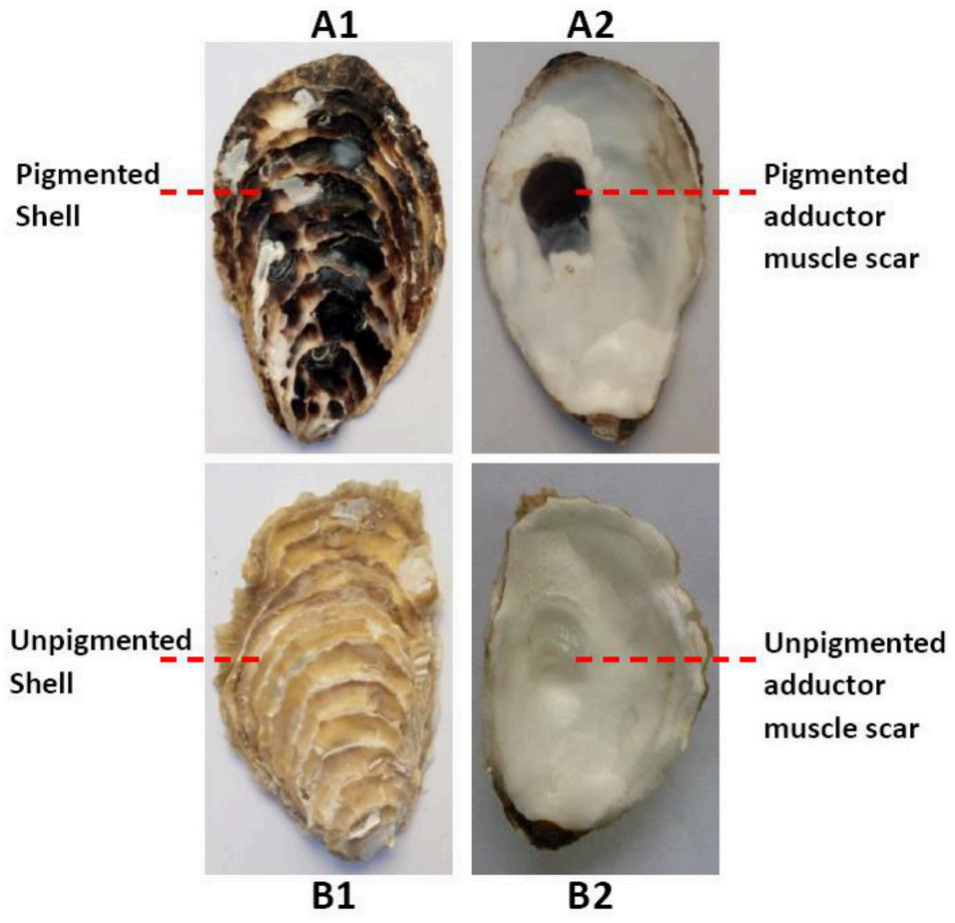
Both the outer shell surfaces **(A1)** and the adductor muscle scars **(A2)** of the oysters were black in the pigmented group; both the outer shell surfaces **(B1)** and the adductor muscle scars **(B2)** of the oysters white in the unpigmented group.

The oysters in RNA-seq experiment, 6–8 cm in shell length, were obtained from a half-sib family (Changdao, Yantai, China). They were dissected and divided into a pigmented group and an unpigmented group, according to both the color of the adductor muscle scars and outer shell surface (Figure 1). Three oysters were included in each group. The adductor muscles and mantles were cut, snap frozen in liquid nitrogen and stored at −80°C until later extraction of RNA. Total RNA was extracted using the guanidinium thiocyanate-phenol-chloroform extraction method (TRIzol, Invitrogen) according to manufacturer's protocol.

The oysters used in real-time Q-PCR experiment were obtained from market (Zhifu, Yantai, China), and divided into pigmented scar VS unpigmented scar and pigmented shell VS unpigmented shell groups. Twelve individuals in each group. The adductor muscles and mantles were cut, snap frozen in liquid nitrogen and stored at −80°C until later extraction of RNA. Total RNA was extracted using TRIzol (Invitrogen) according to manufacturer's protocol.

No specific permissions were required for the above sampling locations. The Pacific oyster is not an endangered or protected species and is not a vertebrate. The oysters used in this study were farmed.

### Pigmentation pattern analysis

The outside surface of each oyster shell was brushed to remove any attached objects and washed to ensure that it was completely clean. The oysters were then boiled for 30 min in a pan on a hot plate. The shells were then opened and the soft bodies removed.

The soft bodies were then placed into an electric blast-drying oven for 12 h. The left shells of the oysters from each farm were marked 1–300 and the right shells 1′–300′ with a pencil. The colors of the outer surface of the shell and the adductor muscle scars of all the oysters were recorded and divided into three types: completely pigmented, partly pigmented, and unpigmented (Brake et al., 2004; Batista et al., 2008). The quantity of oysters whose outer surface of the left shell and left adductor muscle scar differed in color was calculated in each farm; that of oysters whose outer surface of the right shell and right adductor muscle scar differed in color was also done in each farm. The independence of the shell color and the adductor muscle scar color was analyzed through Chi-square test based on the quantities of oysters whose outer surface of the shell and adductor muscle scar in the same side differed in color.

### Correlation between pigmentation and dried soft-body weight

The dried soft bodies of the oysters with an unpigmented or completely pigmented outer shell surface were weighed and recorded, and those with unpigmented or completely pigmented adductor muscle scar were also weighed and recorded.

The correlations between the color of the adductor muscle scar or the outer surface of the shell and the dried soft-body weight were analyzed using unpaired Student's *t*-test in SPSS 20.0. The cut off for significance for the *p*-values was 0.05.

### Comparison of antioxidant physiology indexes between white and black adductor muscles

Twelve white muscles homogenates and 12 black ones were prepared, respectively. Hydroxyl free radical scavenging capacity (HFRSC) of these homogenates were determined based on Fenton reaction (Glick, 2006) using a commercially available detection kit (Nanjing Jiancheng Bioengineering Institute) according to the manufacturer's instructions. The malondialdehyde (MDA) content, the total antioxidant capacity (TAC) and the protein concentration (using the dying method of Coomassie brilliant blue) were also detected with the assay kits from the same company.

### Transcriptome analysis of muscles and mantles with different color

Total RNA was extracted from each of the three “black muscles,” three “white muscles,” three “black mantles,” and three “white mantles.” These three samples combined together comprised the “black muscle,” “white muscle,” “black mantle,” and “white mantle” samples, respectively.

Poly-A RNA was isolated with oligo-dT-coupled beads from 20 μg total RNA from each sample and then sheared. The isolated RNA samples were used for first strand cDNA synthesis, which was performed with random hexamers and Superscript II reverse transcriptase (Invitrogen). The second strand was synthesized with *Escherichia coli* DNA PolI (Invitrogen). Double stranded cDNA was purified with Qiaquick PCR purification kit (Qiagen, Germantown, MD, USA). After end repair and addition of a 3′ dA overhang, the cDNA was ligated to Illumina paired-end adapter oligo mix, and size selected to ~200 bp fragments by gel purification. After 15 PCR cycles the libraries were sequenced using Illumina sequencing platform and the paired-end sequencing module.

The transcriptomes of “black muscle,” “white muscle,” “black mantle,” and “white mantle” were sequenced using 90 bp paired-end RNA-seq. The gene expression levels were determined in different tissues based on their reads per kilobase (RPKM) values of gene model per million mapped reads. After log2 conversion of RPKM values, upregulated and downregulated genes between “black muscle” and “white muscle” or between “black mantle” and “white mantle” were statistically analyzed with *T*-test and false discovery rate (FDR) correction. Differential gene expression was considered significant at an adjusted *P* < 0.01 (Xu et al., 2016; Yang et al., 2016).

Based on functional annotation using Gene Ontology (GO) and Kyoto Encyclopedia of Genes and Genomes (KEGG), we extracted GO terms or KEGG pathways that were enriched in differently expressed genes between “black muscle” and “white muscle” or between “black mantle” and “white mantle” with EnrichPipeline (Chen et al., 2010).

### Validating the differentially expressed genes by real-time quantitative RT-PCR

Because no biological replicates were performed in RNA-seq, the further validation for the RNA-seq results by real-time quantitative RT-PCR were carried out (Xu et al., 2016; Yang et al., 2016). For reverse transcription, the first-strand cDNA was synthesized according to M-MLV RT Usage information (Promega, USA) using oligo (dT)-adaptor (5′-CTCGAGATCGATGCGGCCGCT17-3′) as primer and the DNase I-treated (Promega) total RNA as template. We verified the difference in the expression levels of three tyrosinase genes, two retinol dehydrogenase genes and two Cytochrome P450 genes between the black and white mantles. A constitutive expression gene, ribosomal protein S18 (RS18), was used as endogenous control (Miyamoto and Kajihara, 2005; Wei et al., 2015a,b). All primers used in this assay were listed in Table 1. The SYBR Green real-time PCR assay was carried out in the BIO-RAD CFX Connect™ Real-Time PCR Detection System. Data were analyzed with the CFX Manager™ SoftwareVersion 3.1 (BIO-RAD, USA). The PCR amplification was performed in a 20 μL volume, with 10 μL SYBR Green PCR Master Mix, 2 μL diluted cDNA, 0.8 μL primers (5 μmol/L), and 7.2 μL DEPC-treated water. The thermal profile was 50°C for 2 min and 94°C for 2 min, followed by 40 cycles of 95°C for 5 S, 60°C for 15 S and 72°C for 20 S. The relative expression levels of these genes were analyzed by the 2^−ΔΔCT^ method described previously (Livak and Schmittgen, 2001; Bustin et al., 2009). Statistical analysis was performed by one-way analysis of variance (one-way ANOVA) using SPSS 16.0 statistical software. The *p*-values less than 0.05 were considered statistically significant. The primers used in this experiment were listed as follows (Table 1).

**Table 1:** The primers used in real-time Q-PCR experiment.

**Primer name**	**Sequence (5′ → 3′)**	**Size (nt)**	**Application**
CGI_10011916-F	TGAGGCGGTGGTGGTGC	17	For CGI_10011916 in real-time PCR
CGI_10011916-R	CCACCACCGCCTCACCTTT	19	For CGI_10011916 in real-time PCR
CGI_10012743-F	ATGGCAGGGGCCATGCCCCAGTTGG	25	For CGI_10012743 in real-time PCR
CGI_10012743-R	TCAAGCCCGCTCGCACTC	18	For CGI_10012743 in real-time PCR
CGI_10013418-F	ACCTGGTCCATCAAAGCC	18	For CGI_10013418 in real-time PCR
CGI_10013418-R	ACTCCTGTTCCCTCCTCC	17	For CGI_10013418 in real-time PCR
CGI_10017214-F	ATGGTACGGGACCAACCTTACTTTG	25	For CGI_10017214 in real-time PCR
CGI_10017214-R	AAATAACGGACTCCCTCTGC	20	For CGI_10017214 in real-time PCR
CGI_10021076-F	TGTAAAGGGCGCTAAACT	17	For CGI_10021076 in real-time PCR
CGI_10021076-R	GAAATCCTCCAGGAATGG	17	For CGI_10021076 in real-time PCR
CGI_10026868-F	GAAATCCTCCAGGAATGGTCATACG	25	For CGI_10026868 in real-time PCR
CGI_10026868-R	TGTCCGTGCTGCTCAATCT	19	For CGI_10026868 in real-time PCR
CGI_10026867-F	TCATACGAACGTACTTTTCTCGTTC	25	For CGI_10026867 in real-time PCR
CGI_10026867-R	TGTCCGTGCTGCTCAATCT	19	For CGI_10026867 in real-time PCR
CGI_10011065-F	ATGGGTTCCGTAACGTCCAAGAAAG	25	For CGI_10011065 in real-time PCR
CGI_10011065-R	TCAGCCAGGGTAGTTAGGG	19	For CGI_10011065 in real-time PCR
CGI_10022185-F	AAATCCTAAAGAAGCCATCC	20	For CGI_10022185 in real-time PCR
CGI_10022185-R	GTCCACCAGTTCGTCTATCC	20	For CGI_10022185 in real-time PCR
CGI_10016640-F	ATGAAATTTCTTCACCATGTGTTTG	25	For CGI_10016640 in real-time PCR
CGI_10016640-R	TGGGTGTCGGGATACTCG	18	For CGI_10016640 in real-time PCR
CGI_10011491-F	GGGAACTCTGGTCTTTGT	18	For CGI_10011491 in real-time PCR
CGI_10011491-R	CGGGACAAACGACCCTACA	20	For CGI_10011491 in real-time PCR
CGI_10017766-F	TAGCTTCTAATGCAGGTGT	19	For CGI_10017766 in real-time PCR
CGI_10017766-R	CTTGTATGGCTTTGTCTTC	19	For CGI_10017766 in real-time PCR
CGI_10028005-F	ATGTCGACAGGAGGAATTCAAACAA	25	For CGI_10028005 in real-time PCR
CGI_10028005-R	GCAGTTGTGGCTGGTTTGTG	20	For CGI_10028005 in real-time PCR
RS18-F	GCCATCAAGGGTATCGGTAGAC	22	For internal reference gene in real-time PCR
RS18-R	CTGCCTGTTAAGGAACCAGTCAG	23	For internal reference gene in real-time PCR

### Drawing the location schematic of tyrosinase genes in scaffolds and the hypothetical melanogenesis pathway

We drew the location schematic of the upregulated tyrosinase genes and their neighboring tyrosinase genes in oyster genome scaffolds referred to the database of oyster genes and omics (http://www.oysterdb.com). Based on the pathway of retinol metabolism in animals in KEGG pathway database (http://www.genome.jp/kegg-bin/show_pathway?map00830) and the results of this study, the hypothetical pathway of retinol dehydrogenase genes and Cytochrome P450 genes involved in melanogenesis was proposed.

## Results

### The adductor muscle scars were inconsistent with the corresponding outer surface in color

**Figure 2:**
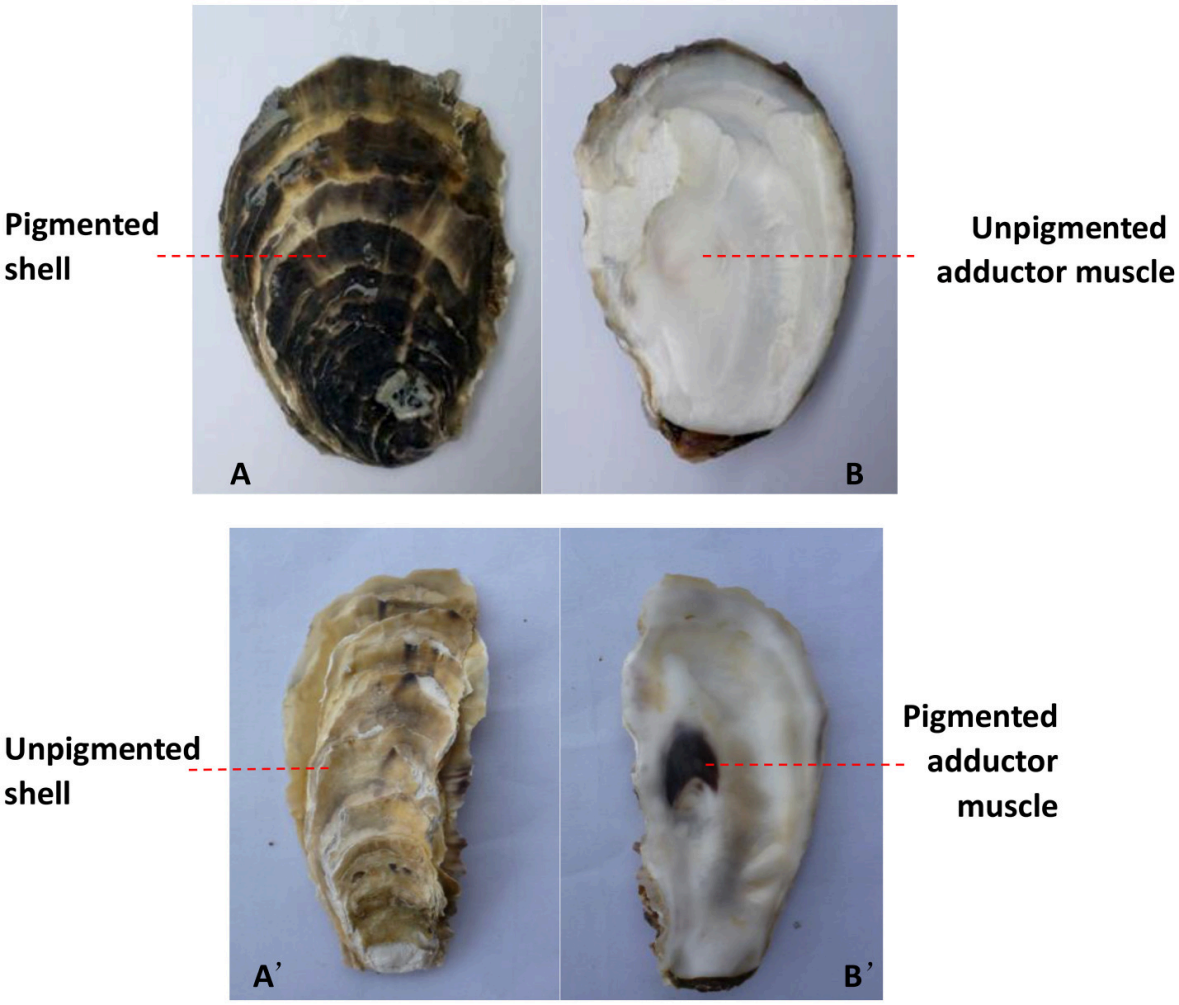
The color differed between the adductor muscle scar and the outer surface of the shell on the same side. The black outer surface of the left shell **(A)** and the white left adductor muscle scar in the same oyster **(B)**; the white outer surface of the right shell **(A')** and the black right adductor muscle scar in the same oyster **(B')**.

The number and percentage of oysters with inconsistent colors of the outer surface of the left shell and the left adductor muscle scar from the three oyster farms are detailed in Table 2 and Table S1, and illustrated using an example in Figures 2A,B. The data show that the color of the outer surface of the left shell was independent of that of the left adductor muscle scar (*P* > 0.05). The number and percentage of oysters with inconsistent colors of the outer surface of the right shell and the right adductor muscle scar from the three oyster farms are detailed in Table 2 and Table S1, and illustrated using an example in Figures 2A',B'. The statistical data show that the color of the outer surface of the right shell was also independent of that of the right adductor muscle scar (*P* > 0.05).

**Table 2:** Number and percentage of oysters whose outer surface of the shell and adductor muscle scar in the same side differed in color.

	**Oyster farm location**		
	**Yantai**	**Rushan**	**Penglai**
Number of oysters whose outer surface of the left shell and left adductor muscle scar differed in color	137	153	154
Percentage	45.67%	51.00%	51.33%
Number of oysters whose outer surface of the right shell and right adductor muscle scar differed in color	137	158	145
Percentage	45.67%	52.67%	48.33%

### The dried soft-body was heavier in oysters with black adductor muscle scars

Though there were three types: completely pigmented, partly pigmented and unpigmented muscle scar or outer shell, but in order to observe the obvious dissimilarity, only the oysters with completely pigmented and unpigmented muscle scar or outer shell were applied to the correlation analysis between pigmentation and soft-body dry weight. Oysters with a black outer shell surface were not found to be heavier in terms of their dried soft-body weight at any of the three farms *(P* > 0.05, Table 3). However, oysters with black left adductor muscle scars were heavier in terms of their dried soft-body weight across all three farms (*P* < 0.05, Table 3); similarly, oysters with black right adductor muscle scars were also heavier in terms of their dried soft-body weight across all three farms (*P* < 0.05, Table 3). Above results hinted that the adductor muscle scar color was correlated with the soft-body dry weight. Thus, the black adductor muscle scar may be one new potential breeding character, which could be used to obtain a larger soft-body dry weight.

**Table 3:** Differences in soft-body dry weight between oysters with black vs. white adductor muscle scar.

	**Oyster farm location**	**Soft-body dry weight (g)**		***P*-value**
		**White (Number of oysters): Mean ± SE**	**Black (Number of oysters): Mean ± SE**	
White vs. black left outer shell surface	Yantai	(*n* = 57): 9.2 ± 0.285	(*n* = 39): 9.8 ± 0.262	*P* = 0.743
	Rushan	(*n* = 125): 8.46 ± 0.133	(*n* = 29): 8.55 ± 0.204	*P* = 0.776
	Penglai	(*n* = 89): 8.36 ± 0.128	(*n* = 60): 8.38 ± 0.147	*P* = 0.909
White vs. black right outer shell surface	Yantai	(*n* = 71): 9.31 ± 0.223	(*n* = 44): 9.51 ± 0.261	*P* = 0.583
	Rushan	(*n* = 101): 8.46 ± 0.113	(*n* = 52): 8.52 ± 0.177	*P* = 0.742
	Penglai	(*n* = 78): 8.41 ± 0.129	(*n* = 69): 8.36 ± 0.123	*P* = 0.784
White vs. black left adductor muscle scar	Yantai	(*n* = 57): 0.75 ± 0.036	(*n* = 39): 0.96 ± 0.057	*P* = 0.002
	Rushan	(*n* = 125): 0.86 ± 0.037	(*n* = 29): 1.12 ± 0.092	*P* = 0.030
	Penglai	(*n* = 89): 1.00 ± 0.042	(*n* = 60): 1.36 ± 0.058	*P* = 0.000
White vs. black right adductor muscle scar	Yantai	(*n* = 71): 0.80 ± 0.041	(*n* = 44): 0.95 ± 0.059	*P* = 0.046
	Rushan	(*n* = 101): 0.85 ± 0.034	(*n* = 52): 1.07 ± 0.070	*P* = 0.005
	Penglai	(*n* = 78): 1.01 ± 0.040	(*n* = 69): 1.32 ± 0.048	*P* = 0.000

*SE denotes standard error*.

### The black adductor muscles may have the stronger HFRSC

**Figure 3:**
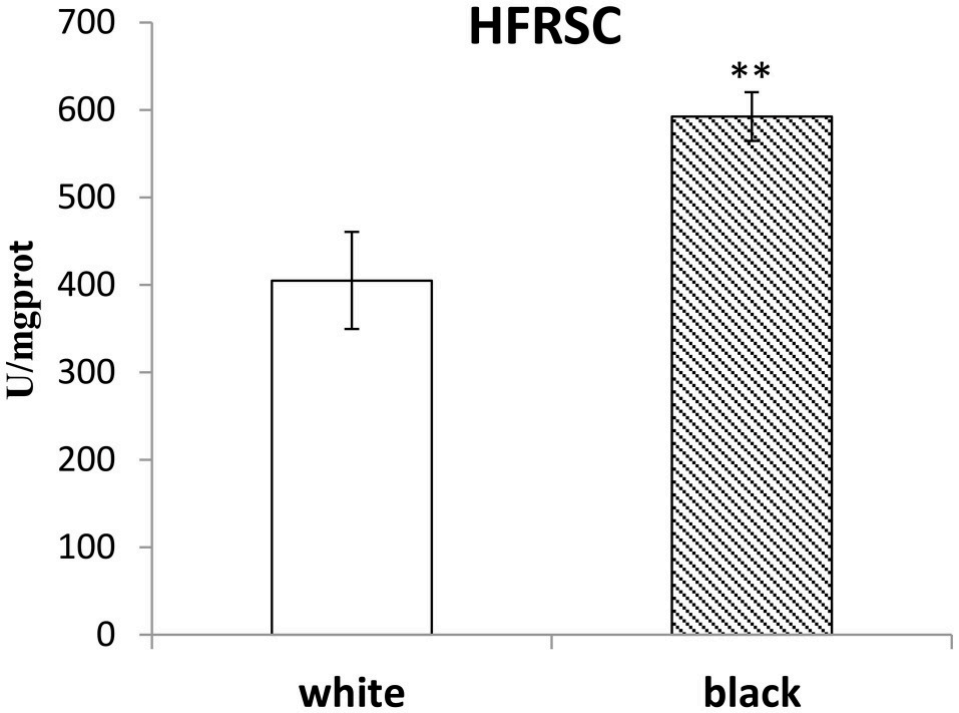
The HFRSC in the white muscles compared to those in the black muscles. The double-asterisk (^**^) indicates statistically significant difference (*P* < 0.01).

The HFRSC, MDA, and TAC in the homogenates of white or black muscles were detected and compared. As shown in Figure 3 and Table 4, the HFRSC in black muscles was higher than that in white ones (*p* < 0.01), but the MDA content and the TAC content were found no different between the black muscles and the white ones.

**Table 4:** The statistical results of HFRSC, MDA, and TAC in the white or black muscles.

	**White adductor muscle (*n* = 12)**	**Black adductor muscle (*n* = 12)**	***P*-value**
HFRSC(U/mgprot)	404.9828 ± 55.4888	592.6644 ± 27.9581	0.0080
TAC(U/mgprot)	1.2484 ± 0.0906	1.2669 ± 0.1755	0.9268
MDA(nmol/mgpro)	4.1515 ± 0.6257	4.2371 ± 0.6011	0.9223

### Differently expressed genes between “black muscle” and “white muscle” or between “black mantle” and “white mantle”

The four *C. gigas* transcriptomes of black muscle, white muscle, black mantle, and white mantle were constructed and sequenced using Illumina Hiseq 2500, and the raw data were submitted to the NCBI SRA database with accession numbers of SRR5638635 (black muscle), SRR5638636 (white muscle), SRR5638638 (black mantle), and SRR5638651 (white mantle). Then, the differentially expressed genes in black VS white muscle and in black VS white mantle were listed in Tables S2, S3, respectively. GO terms enriched based on the upregulated genes in “black muscle” compared to “white muscle” are presented in Table S4. GO terms enriched based on the upregulated genes in “black mantle” compared to “white mantle” are presented in Table S5. The results showed that the enriched GO terms related to adductor muscle scar pigmentation were different to those related to outer shell surface pigmentation.

**Figure 4:**
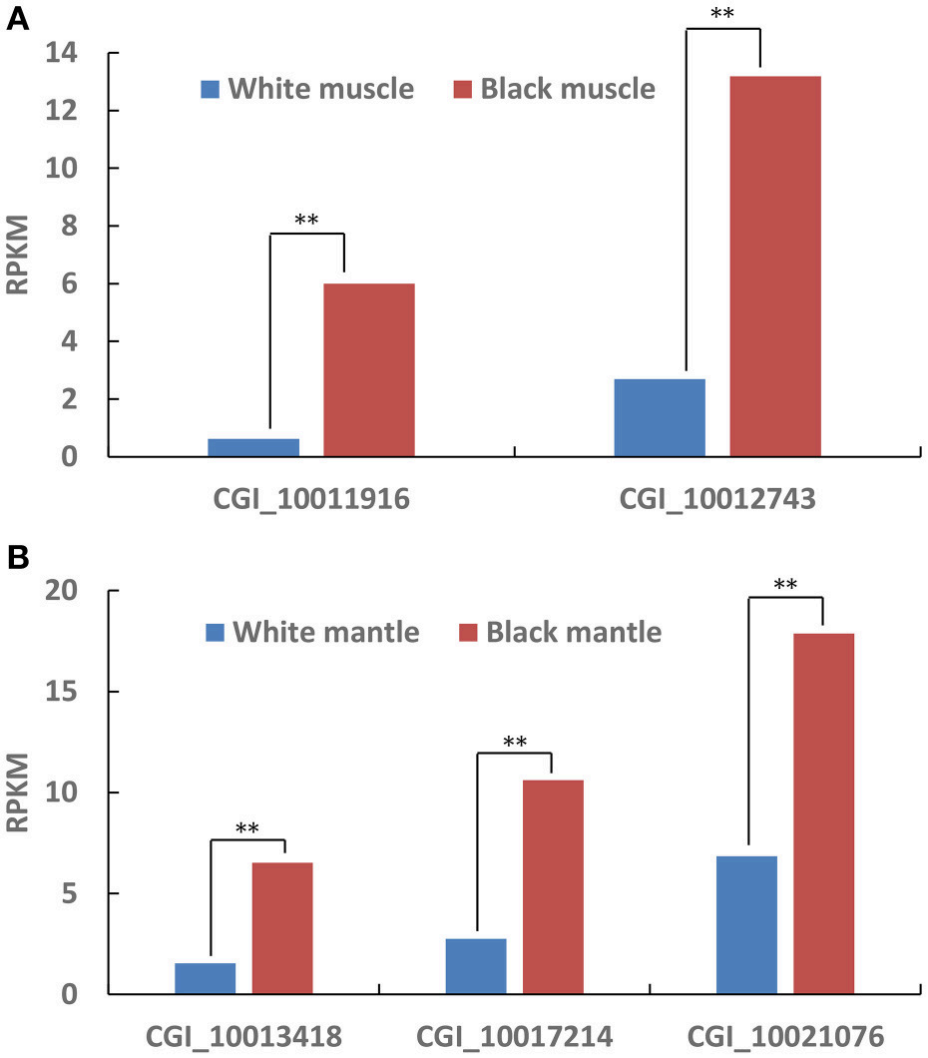
The upregulated tyrosinase genes in black muscle compared to white muscle **(A)** and in black mantle compared to white mantle **(B)**. The double-asterisk (^**^) indicates statistically significant difference (*P* < 0.01).

According to the Tables S2, S3, the differentially expressed tyrosinase genes only existed in the Up-Regulation gene sets of black_Amu/white_Amu or black_Man/white_Man. Other trosinase genes weren't differentially expressed between the “black muscle” and the “white muscle,” or between the “black mantle” and the “white mantle.” We found that two tyrosinase genes (CGI_10011916 and CGI_10012743) were significantly upregulated in the “black muscle” compared to the “white muscle” (Figure 4A; Table 5). We also found that three tyrosinase genes (CGI_10013418, CGI_10017214 and CGI_10021076) were significantly upregulated in the “black mantle” compared to the “white mantle” (Figure 4B; Table 5).

**Table 5:** The upregulated tyrosinase genes in black muscle compared to white muscle and in black mantle compared to white mantle.

	**GeneID**	**White**	**Black**	***P*-value**	**FDR**
**Muscle**	CGI_10011916	0.616480559	6.001048452	2.27E-07	2.39E-06
	CGI_10012743	2.687525191	13.18702621	2.43E-10	3.71E-09
**Mantle**	CGI_10013418	1.541272631	6.521416701	2.54E-05	4.21E-04
	CGI_10017214	2.747091807	10.60641294	4.09E-07	9.82E-06
	CGI_10021076	6.850485317	17.86798799	3.19E-07	7.81E-06

As presented in Table S7, Cytochrome P450 genes were found upregulated not only in the black muscle (CGI_10016640, CGI_10011491, CGI_10017766, and CGI_10028005) and but also in the black mantles (CGI_10011065 and CGI_10022185). We noted that the upregulated cytochrome P450 genes in black muscle were also different with ones in black mantle.

We found the “Retinol metabolism” pathway enriched based on the upregulated genes in “black muscle” compared to “white muscle” (*P* < 0.01), and also found the same pathway enriched based on the upregulated genes in “black mantle” compared to “white mantle” (*P* < 0.01), but the upregulated genes in the above two groups were complete different (Table S7). In the muscle group, the upregulated genes were Cytochrome P450 1A2, Cytochrome P450 1A2, Cytochrome P450 26A1, Cytochrome P450 3A24, 17-beta-hydroxysteroid dehydrogenase 13 and Retinoic acid receptor responder protein 3, but in the mantle group, those were Cytochrome P450 1A1, Retinal dehydrogenase 1, Retinal dehydrogenase 1, Cytochrome P450 3A29, Diacylglycerol O-acyltransferase 1, and Omega-crystallin.

### The co-expression of tyrosinase genes didn't locate in the same gene cluster

**Figure 5:**
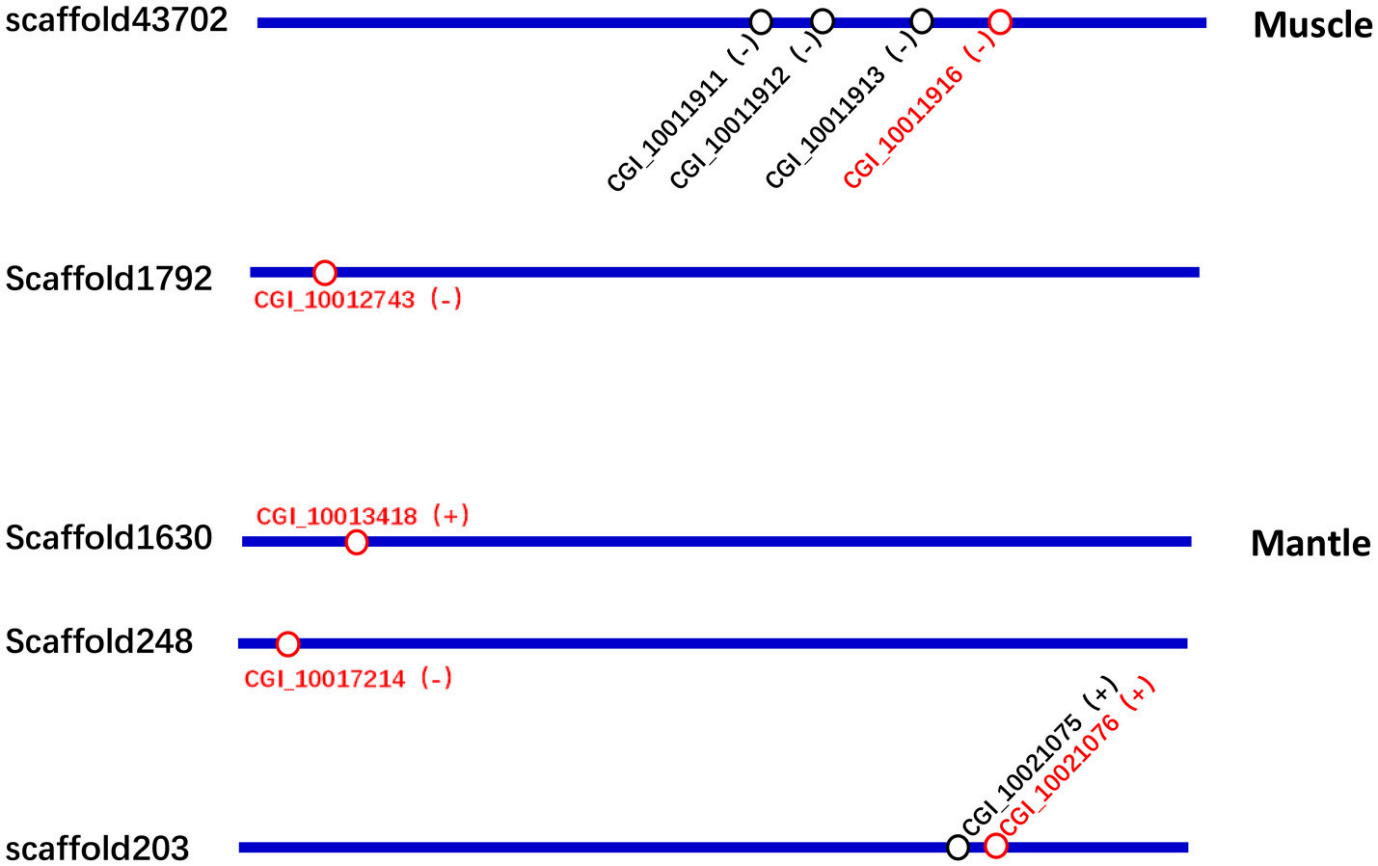
The location schematic of the upregulated tyrosinase genes (red) and their neighboring tyrosinase genes (black) in scaffolds.

As shown in Figure 5 and Table S6, the two upregulated tyrosinase genes in the “black muscle,” CGI_10011916 and CGI_10012743, were located in scaffold43702 and scaffold1792, respectively; the three upregulated tyrosinase genes in the “black mantle,” CGI_10013418, CGI_10017214, and CGI_10021076, were located in scaffold1630, scaffold248, and scaffold203, respectively. Therein CGI_10011916 was among one tyrosinase gene cluster, CGI_10021076 among another one (Figure 5 and Table S6).

### Validation of RNAseq results by qRT-PCR

Based on the results of RNAseq, three tyrosinase genes, two retinol dehydrogenase genes, and two Cytochrome P450 genes were selected to perform qRT-PCR in black and white mantles. It was found that all the seven genes were upregulated in the “black mantle” compared to the “white mantle” (Table 6, Figure S1). Especially, two tyrosinase genes (CGI_10013418 and CGI_10021076), two retinol dehydrogenase genes (CGI_10026867 and CGI_10026868), and one Cytochrome P450 gene were significantly upregulated.

**Table 6:** The expression levels of Tyrosinase, Retinal dehydrogenase, and Cytochrome P450 genes in the black or white mantles.

**ID**	**Name**	**White mantle (*n* = 12)**	**Black mantle (*n* = 12)**	***P*-value**
CGI_10013418	Putative tyrosinase-like protein tyr-1	0.680937 ± 0.285112	1.175346 ± 0.541746	0.013723
CGI_10017214	Putative tyrosinase-like protein tyr-3	1.089366 ± 0.521610	1.147055 ± 0.599798	0.812011
CGI_10021076	Putative tyrosinase-like protein tyr-3	0.606336 ± 0.374803	1.156025 ± 0.560302	0.012947
CGI_10026867	Retinal dehydrogenase 1	0.567329 ± 0.483198	1.313677 ± 0.820115	0.016328
CGI_10026868	Retinal dehydrogenase 1	0.700355 ± 0.277495	1.172389 ± 0.648519	0.037079
CGI_10011065	Cytochrome P450 1A1	1.199263 ± 0.513051	1.275978 ± 0.694909	0.771093
CGI_10022185	Cytochrome P450 3A29	0.736789 ± 0.143924	1.080865 ± 0.447417	0.023813

We also decided the expression levels of two tyrosinase genes, two retinol dehydrogenase genes and four Cytochrome P450 genes by qRT-PCR in the black and white adductor muscles. It was found that the eight genes were upregulated in the “black adductor muscle” compared to the “white adductor muscle” (Table 7, Figure S2). Especially, two tyrosinase genes (CGI_10011916 and CGI_10012743), and two Cytochrome P450 genes (CGI_10011491 and CGI_10017766) were significantly upregulated.

**Table 7:** The expression levels of Tyrosinase, Retinal dehydrogenase, and Cytochrome P450 genes in the black or white adductor muscles.

**ID**	**Name**	**White adductor muscle (*n* = 12)**	**Black adductor muscle (*n* = 12)**	***P*-value**
CGI_10011916	Putative tyrosinase-like protein tyr-3	0.905628 ± 0.340346	1.386220 ± 0.564022	0.024248
CGI_10012743	Putative tyrosinase-like protein tyr-3	0.415715 ± 0.219569	1.293165 ± 0.650154	0.000335
CGI_10026867	Retinal dehydrogenase 1	0.041847 ± 0.030044	0.070180 ± 0.049170	0.117169
CGI_10026868	Retinal dehydrogenase 1	1.070690 ± 0.448060	1.191031 ± 0.605755	0.601609
CGI_10016640	Cytochrome P450 1A2	1.053898 ± 0.572561	1.152941 ± 0.606137	0.697402
CGI_10011491	Cytochrome P450 1A2	0.676692 ± 0.313043	1.091032 ± 0.455515	0.020985
CGI_10017766	Cytochrome P450 26A1	0.673012 ± 0.388850	1.154980 ± 0.555872	0.027775
CGI_10028005	Cytochrome P450 3A24	0.994803 ± 0.516684	1.040756 ± 0.563629	0.843838

A comparison of qRT-PCR and RNA-seq data of the five tyrosinase genes was conducted. The results showed that the expression patterns of CGI_10011916, CGI_10012743 in muscle and CGI_10013418 and CGI_10021076 in mantle agreed well between RNA-seq and qRT-PCR, and CGI_10017214 had a similar trend in change of expression pattern between RNA-seq and qRT-PCR (Table 8).

**Table 8:** Comparison of qRT-PCR data and RNA-seq data for five tyrosinase genes.

	**Gene**	**qRT-PCR the expression ratio 2^−ΔΔCT^**			**RNA-seq log_2_(fold_change)**		
		**White**	**Black**	***P*-value**	**White**	**Black**	***P*-value**
Muscle	CGI_10011916	0.905628	1.386220	0.024248	0.616480559	6.001048452	2.27E-07
	CGI_10012743	0.415715	1.293165	0.000335	2.687525191	13.18702621	2.43E-10
Mantle	CGI_10013418	0.680937	1.175346	0.013723	1.541272631	6.521416701	2.54E-05
	CGI_10017214	1.089366	1.147055	0.812011	2.747091807	10.60641294	4.09E-07
	CGI_10021076	0.606336	1.156025	0.012947	6.850485317	17.86798799	3.19E-07

## Discussion

### The probable physiological base for the oysters with black adductor muscle scars have heavier dried soft-body

It has been studied that the water-soluble melanin fractions from squid ink was more efficient than carnosine as the free radical scavenger (Xiao yan et al., 2003; Vate and Benjakul, 2013; Guo et al., 2014). The black muscles may contain more melanin that could scavenge hydroxyl free radicals. Bivalve adductor muscles are composed of semi-translucent and white opaque muscles. The former is thought to be responsible for the quick closure of shells, and the latter for catch contraction that keeps shells tightly closed for many hours and maintains this tension for long periods with little energy consumption (Funabara et al., 2013).

According to common sense, the shells of dead oyster is completely open because of the relaxation of its muscle, which should need the least energy consumption. So, it is possible that the physiological cost of maintaining shells open at an intermediate position should be larger and the physiological metabolization in the adductor muscle is likely to be intensive when oysters open their shells to filter algae. This would result in the production of many free radicals that could impair the muscle cells of oyster, just as human (Konczol et al., 1998; Kerksick and Zuhl, 2015; Pal et al., 2017). Adductor muscles attached to black adductor muscle scars might contain melanin that could eliminate these free radicals and reduce the damage to the adductor muscle (Sarna et al., 1986; Korytowski et al., 1987; Rozanowska et al., 1999). Compared with oysters with white adductor muscle scars, those with black scars might enable them to open their shells for longer and filter more algae, which could promote an accelerated growth rate.

### The different members of the same gene families responsible for the independence of pigmentation processes in oyster different organs

It has been found that the pigmented adductor muscle scar to which the “black muscle” was attached contained melanin (Hao et al., 2015) and a tyrosinase gene was involved in melanin production (Kumar et al., 2011). So, the results of this study suggests that these two tyrosinase genes may play an important role in melanin deposition on adductor muscle scar in *C. gigas*. Adductor muscle scar may not only function to open/close the shell, but also to produce melanin. Previous work has shown that the pigmented shell, to which the “black mantle” corresponds, also contains melanin (Yu et al., 2015), and tyrosinase genes are expressed in bivalve mantles (Zhang et al., 2012; Aguilera et al., 2014), but didn't correlate gene expression with melanin deposition in mollusks. However, the results of this study hinted that these three tyrosinase genes were likely involved in melanin deposition on the outer surface of *C. gigas* shells.

Two steps of melanin synthesis are catalyzed by tyrosinase (Goodwill et al., 1998); therefore, tyrosinase is the rate-limiting enzyme in the production of melanin (Sanchez-Ferrer et al., 1995). It was easy to understand that the difference in pigmentation of the adductor muscle scars or the outer surfaces of the shell could be due to the differential expression of the genes encoding tyrosinase. However, we noted that the differentially expressed tyrosinase genes in the up-regulation gene set of black muscle/white muscle were completely different with those of black mantle/white mantle, hinting that the different members of the tyrosinase gene family (Zhang et al., 2012; Yu et al., 2015) could play a role in the independent pigmentation of different organs.

It has usually been observed that the co-expression of neighboring genes occurred in gene cluster (Boutanaev et al., 2002; Lercher et al., 2002, 2003; Birnbaum et al., 2003; Fukuoka et al., 2004). But, the co-expression of neighboring tyrosinase genes wasn't observed in this study, on the contrary, the non-neighboring tyrosinase genes co-expressed, hinting that the co-expression of these non-neighboring tyrosinase genes was probably caused by the requirement of physiologic function not the adjacent location in genome.

Different members of Cytochrome P450 gene family were found upregulated not only in the black muscle (CGI_10016640, CGI_10011491, CGI_10017766, and CGI_10028005) and but also in the black mantles (CGI_10011065 and CGI_10022185). The upregulated cytochrome P450 genes in black muscle were also different with ones in black mantle, and Cytochrome P450 can involve in the melanin biosynthesis in *Streptomyces griseus* (Funa et al., 2005), which hinted that the different members of Cytochrome P450 gene family were also involved in the independent pigmentation of different organs of oyster.

Neuroglobin and cytoglobin were two members belonging to the vertebrate globin superfamily, and they had similar physiologic function (oxygen transport and storage) in different organs (Burmester et al., 2004). In this study, it was also found that the different members of the same gene family performed the same function (pigmentation) in different organs of oyster.

### Retinol metabolism pathway may involve in melanin formation of oyster

Retinol (Vitamin A1) is one of the animal forms of vitamin A and retinol dehydrogenases can transfer vitamin A into acidum vitamin A (Liden and Eriksson, 2006). Acidum vitamin A (also called as retinoic acid) could induce the melanocyte maturation and promote basal levels of melanogenesis (Lotan and Lotan, 1980) by increasing the tyrosinase activity (Li and Zhu, 2001), it has also been reported that retinoic acid is critical in establishing asymmetric pigmentation of flatfish (Shao et al., 2017). Cytochrome P450 can also involve in the melanin biosynthesis by catalyzing retinoate into retinoic acid (Funa et al., 2005). The more important was that the “Retinol metabolism” pathway was enriched based on the upregulated genes in “black muscle” compared to “white muscle” or in “black mantle” compared to “white mantle.”

**Figure 6:**
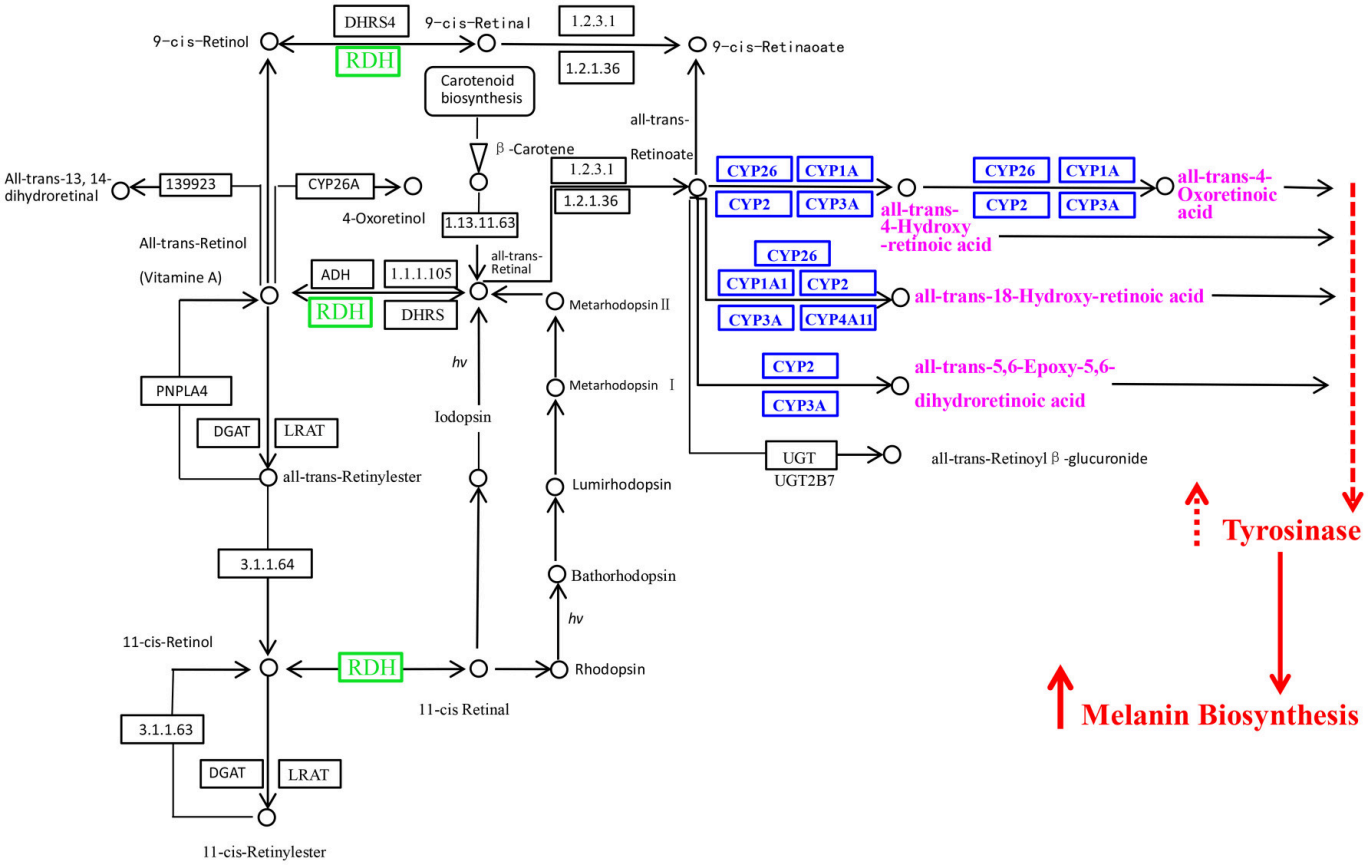
The retinol dehydrogenase genes and Cytochrome P450 genes in the retinol metabolism pathway and their probable associations with melanin biosynthesis. RDH, retinol dehydrogenase; CYP, Cytochrome P450. The picture was got based on the pathway of retinol metabolism in animals in KEGG pathway database (http://www.genome.jp/kegg-bin/show_pathway?map00830) and the results of this study.

Combining the results of this study and the pathway information of “retinol metabolism in animals” acquired from KEGG pathway database (http://www.genome.jp/kegg-bin/show_pathway?map00830), we proposed the hypothetical pathway of retinol dehydrogenase genes and Cytochrome P450 genes involved in melanin biosynthesis of oyster (Figure 6). In this hypothetical pathway, retinal dehydrogenase and Cytochrome P450 affected retinoic acid production, more retinoic acid increased the tyrosinase activity, thus more melanin was generated.

In conclusion, it was found that the pigmentation of oyster adductor muscle scar was unrelated with that of its corresponding shell outer surface; interestingly, a relationship between the color of the adductor muscle scars and the dried soft-body weight; the different members of the tyrosinase or Cytochrome P450 gene families could play a role in the independent pigmentation of different organs. These findings could provide some suggestions for further pigmentation study of mollusks.

## Author contributions

XW conceived and designed the experiments. WY, CH, ZC and NW performed the experiments. LW, JC, QJ and FX analyzed the data. ZL and WG contributed reagents/materials/analysis tools. XW, WY and CH wrote the paper. All authors reviewed the manuscript.

### Conflict of interest statement

The authors declare that the research was conducted in the absence of any commercial or financial relationships that could be construed as a potential conflict of interest.

## References

[B1] Aguilera F. McDougall C. Degnan B. M. (2014). Evolution of the tyrosinase gene family in bivalve molluscs: independent expansion of the mantle gene repertoire. Acta Biomater. 10, 3855–3865. 10.1016/j.actbio.2014.03.031 24704693

[B2] Batista F. M. Ben-Hamadou R. Fonseca V. G. Taris N. Ruano F. Reis-Henriques M. A. (2008). Comparative study of shell shape and muscle scar pigmentation in the closely related cupped oysters *Crassostrea angulata, C. gigas* and their reciprocal hybrids. Aquat. Living Resour. 21, 31–38. 10.1051/alr:2008019

[B3] Birnbaum K. Shasha D. E. Wang J. Y. Jung J. W. Lambert G. M. Galbraith D. W.. (2003). A gene expression map of the Arabidopsis root. Science 302, 1956–1960. 10.1126/science.1090022 14671301

[B4] Boutanaev A. M. Kalmykova A. I. Shevelyov Y. Y. Nurminsky D. I. (2002). Large clusters of co-expressed genes in the Drosophila genome. Nature 420, 666–669. 10.1038/nature01216 12478293

[B5] Brake J. Evans F. Langdon C. (2004). Evidence for genetic control of pigmentation of shell and mantle edge in selected families of Pacific oysters, *Crassostrea gigas*. Aquaculture 229, 89–98. 10.1016/S0044-8486(03)00325-9

[B6] Burmester T. Haberkamp M. Mitz S. Roesner A. Schmidt M. Ebner B.. (2004). Neuroglobin and cytoglobin: genes, proteins and evolution. IUBMB Life 56, 703–707. 10.1080/15216540500037257 15804835

[B7] Bustin S. A. Benes V. Garson J. A. Hellemans J. Huggett J. Kubista M.. (2009). The MIQE guidelines: minimum information for publication of quantitative real-time PCR experiments. Clin. Chem. 55, 611–622. 10.1373/clinchem.2008.112797 19246619

[B8] Chen S. Yang P. Jiang F. Wei Y. Ma Z. Kang L. (2010). *De novo* analysis of transcriptome dynamics in the migratory locust during the development of phase traits. PLoS ONE 5:e15633. 10.1371/journal.pone.0015633 21209894 PMC3012706

[B9] Feng D. Li Q. Yu H. Zhao X. Kong L. (2015). Comparative transcriptome analysis of the Pacific oyster *Crassostrea gigas* characterized by shell colors: identification of genetic bases potentially involved in pigmentation. PLoS ONE 10:e0145257. 10.1371/journal.pone.0145257 26693729 PMC4691203

[B10] Fukuoka Y. Inaoka H. Kohane I. S. (2004). Inter-species differences of co-expression of neighboring genes in eukaryotic genomes. BMC Genomics 5:4. 10.1186/1471-2164-5-4 14718066 PMC331401

[B11] Funa N. Funabashi M. Ohnishi Y. Horinouchi S. (2005). Biosynthesis of hexahydroxyperylenequinone melanin via oxidative aryl coupling by cytochrome P-450 in *Streptomyces griseus*. J. Bacteriol. 187, 8149–8155. 10.1128/JB.187.23.8149-8155.2005 16291687 PMC1291289

[B12] Funabara D. Watanabe D. Satoh N. Kanoh S. (2013). Genome-wide survey of genes encoding muscle proteins in the pearl oyster, *Pinctada fucata*. Zool. Sci. 30, 817–825. 10.2108/zsj.30.817 24125646

[B13] Ge J. Li Q. Yu H. Kong L. (2015). Mendelian inheritance of golden shell color in the Pacific oyster *Crassostrea gigas*. Aquaculture 441, 21–24. 10.1016/j.aquaculture.2015.01.031

[B14] Glick D. (2006). Methods for the Measurement of Hydroxyl Radicals in Biochemical Systems: Deoxyribose Degradation and Aromatic Hydroxylation. New York, NY: John Wiley & Sons, Inc. 10.1002/9780470110546.ch2 2833681

[B15] Goodwill K. E. Sabatier C. Stevens R. C. (1998). Crystal structure of tyrosine hydroxylase with bound cofactor analogue and iron at 2.3 Å resolution: self-hydroxylation of Phe300 and the Pterin-binding site. Biochemistry 37, 13437–13445. 10.1021/bi981462g 9753429

[B16] Guo X. Chen S. Hu Y. Li G. Liao N. Ye X.. (2014). Preparation of water-soluble melanin from squid ink using ultrasound-assisted degradation and its anti-oxidant activity. J. Food Sci. Technol. 51, 3680–3690. 10.1007/s13197-013-0937-7 25477634 PMC4252405

[B17] Hao S. Hou X. Wei L. Li J. Li Z. Wang X. (2015). Extraction and identification of the pigment in the adductor muscle scar of pacific oyster *Crassostrea gigas*. PLoS ONE 10:e0142439. 10.1371/journal.pone.0142439 26555720 PMC4640808

[B18] Higuera-Ruiz R. Elorza J. (2011). Shell thickening and chambering in the oyster *Crassostrea gigas*: natural and anthropogenic influence of tributyltin contamination. Environ. Technol. 32, 583–591. 10.1080/09593330.2010.506201 21877539

[B23] Kerksick C. M. Zuhl M. (2015). Mechanisms of oxidative damage and their impact on contracting muscle, in Antioxidants in Sport Nutrition, ed Lamprecht M. (Boca Raton, FL: CRC Press), 1–16. 26065093

[B19] Kollias N. Sayre R. M. Zeise L. Chedekel M. R. (1991). New trends in photobiology: photoprotection by melanin. J. Photochem. Photobiol. B 9, 135–160. 10.1016/1011-1344(91)80147-A 1907647

[B20] Konczol F. Lorinczy D. Belagyi J. (1998). Effect of oxygen free radicals on myosin in muscle fibres. FEBS Lett. 427, 341–344. 10.1016/S0014-5793(98)00439-6 9637253

[B21] Korytowski W. Pilas B. Sarna T. Kalyanaraman B. (1987). Photoinduced generation of hydrogen peroxide and hydroxyl radicals in melanins. Photochem. Photobiol. 45, 185–190. 10.1111/j.1751-1097.1987.tb05362.x 3031709

[B22] Kumar C. M. Sathisha U. V. Dharmesh S. Rao A. G. Singh S. A. (2011). Interaction of sesamol (3,4-methylenedioxyphenol) with tyrosinase and its effect on melanin synthesis. Biochimie 93, 562–569. 10.1016/j.biochi.2010.11.014 21144881

[B24] Lee S. W. Jang Y. N. Kim J. C. (2011). Characteristics of the aragonitic layer in adult oyster shells, *Crassostrea gigas*: structural study of myostracum including the adductor muscle scar. Evid. Based Complement. Alternat. Med. 2011:742963. 10.1155/2011/742963 21716680 PMC3118482

[B25] Lercher M. J. Blumenthal T. Hurst L. D. (2003). Coexpression of neighboring genes in *Caenorhabditis elegans* is mostly due to operons and duplicate genes. Genome Res. 13, 238–243. 10.1101/gr.553803 12566401 PMC420373

[B26] Lercher M. J. Urrutia A. O. Hurst L. D. (2002). Clustering of housekeeping genes provides a unified model of gene order in the human genome. Nat. Genet. 31, 180–183. 10.1038/ng887 11992122

[B27] Li H. Zhu W. (2001). Effects of acidum vitamin A on tyrosinase activity. Chin. Med. J. 114, 415–417. 11780467

[B28] Li N. Hu J. Wang S. Cheng J. Hu X. Lu Z. (2010). Isolation and identification of the main carotenoid pigment from the rare orange muscle of the Yesso scallop. Food Chem. 118, 616–619. 10.1016/j.foodchem.2009.05.043

[B29] Liden M. Eriksson U. (2006). Understanding retinol metabolism: structure and function of retinol dehydrogenases. J. Biol. Chem. 281, 13001–13004. 10.1074/jbc.R500027200 16428379

[B30] Liu H. Zheng H. Zhang H. Deng L. Liu W. Wang S.. (2015). A *de novo* transcriptome of the noble scallop, *Chlamys nobilis*, focusing on mining transcripts for carotenoid-based coloration. BMC Genomics 16:44. 10.1186/s12864-015-1241-x 25651863 PMC4342821

[B31] Livak K. J. Schmittgen T. D. (2001). Analysis of relative gene expression data using real-time quantitative PCR and the 2(-Delta Delta C(T)) Method. Methods 25, 402–408. 10.1006/meth.2001.1262 11846609

[B32] Lotan R. Lotan D. (1980). Stimulation of melanogenesis in a human melanoma cell line by retinoids. Cancer Res. 40, 3345–3350. 6253061

[B33] Maiani G. Caston M. J. Catasta G. Toti E. Cambrodon I. G. Bysted A.. (2009). Carotenoids: actual knowledge on food sources, intakes, stability and bioavailability and their protective role in humans. Mol. Nutr. Food Res. 53(Suppl. 2), S194–S218. 10.1002/mnfr.200800053 19035552

[B34] Maoka T. (2011). Carotenoids in marine animals. Mar. Drugs 9, 278–293. 10.3390/md9020278 21566799 PMC3093257

[B35] Maoka T. Kuwahara T. Narita M. (2014). Carotenoids of sea angels *Clione limacina* and *Paedoclione doliiformis* from the perspective of the food chain. Mar. Drugs 12, 1460–1470. 10.3390/md12031460 24633249 PMC3967221

[B36] Miyamoto H. Kajihara K. (2005). Conserved ribosomal protein sequences S5, S18, S27, S30 in the Pacific oyster Crassostrea gigas and Crassostrea virginica. Memoirs of the School of Biology-Oriented Science and Technology of Kinki University 16, 1–5.

[B37] Pal S. Chaki B. Chattopadhyay S. Bandyopadhyay A. (2017). High intensity exercise induced oxidative stress and skeletal muscle damage in post-pubertal boys and girls: a comparative study. J. Strength Cond. Res. [Epub ahead of print]. 10.1519/JSC.0000000000002167 28767482

[B38] Rao A. V. Rao L. G. (2007). Carotenoids and human health. Pharmacol. Res. 55, 207–216. 10.1016/j.phrs.2007.01.012 17349800

[B39] Rozanowska M. Sarna T. Land E. J. Truscott T. G. (1999). Free radical scavenging properties of melanin interaction of eu- and pheo-melanin models with reducing and oxidising radicals. Free Radical. Bio. Med. 26, 518–525. 10218640 10.1016/s0891-5849(98)00234-2

[B40] Sanchez-Ferrer A. Rodriguez-Lopez J. N. Garcia-Canovas F. Garcia-Carmona F. (1995). Tyrosinase: a comprehensive review of its mechanism. Biochim. Biophys. Acta 1247, 1–11. 10.1016/0167-4838(94)00204-T 7873577

[B41] Sanford E. Markd C. Christopherj L. (2009). Heritability of shell pigmentation in the Pacific oyster, *Crassostrea gigas*. Aquaculture 286, 211–216. 10.1016/j.aquaculture.2008.09.022

[B42] Sarna T. Pilas B. Land E. J. Truscott T. G. (1986). Interaction of radicals from water radiolysis with melanin. Biochim. Biophys. Acta 883, 162–167. 10.1016/0304-4165(86)90147-9 3015231

[B43] Shao C. Bao B. Xie Z. Chen X. Li B. Jia X.. (2017). The genome and transcriptome of Japanese flounder provide insights into flatfish asymmetry. Nat. Genet. 49, 119–124. 10.1038/ng.3732 27918537

[B44] Sharma S. Wagh S. Govindarajan R. (2002). Melanosomal proteins–role in melanin polymerization. Pigment Cell Res. 15, 127–133. 10.1034/j.1600-0749.2002.1o076.x 11936270

[B45] Vate N. K. Benjakul S. (2013). Antioxidative activity of melanin-free ink from splendid squid (*Loligo formosana*). Int. Aquat. Res. 5, 1–12. 10.1186/2008-6970-5-9

[B46] Wan S. Li Q. Liu T. Yu H. Kong L. (2017). Heritability estimates for shell color-related traits in the golden shell strain of Pacific oyster (*Crassostrea gigas*) using a molecular pedigree. Aquaculture 476, 65–71. 10.1016/j.aquaculture.2017.04.012

[B47] Wei L. Wang Q. Ning X. Mu C. Wang C. Cao R.. (2015a). Combined metabolome and proteome analysis of the mantle tissue from Pacific oyster *Crassostrea gigas* exposed to elevated pCO2. Comp. Biochem. Physiol. Part D Genomics Proteomics 13, 16–23. 10.1016/j.cbd.2014.12.001 25559488

[B48] Wei L. Wang Q. Wu H. Ji C. Zhao J. (2015b). Proteomic and metabolomic responses of Pacific oyster *Crassostrea gigas* to elevated pCO2 exposure. J. Proteomics 112, 83–94. 10.1016/j.jprot.2014.08.010 25175059

[B49] Williams S. T. (2017). Molluscan shell colour. Biol. Rev. Camb. Philos. Soc. 92, 1039–1058. 10.1111/brv.12268 27005683

[B50] Xiao yan L. I. Liu Z. H. Cai R. X. (2003). A Study on Scavenging activity of melanin to hydroxyl free radicals. J. Sichuan Univ. 40, 1132–1136.

[B51] Xu Z. Li T. Li E. Chen K. Ding Z. Qin J. G.. (2016). Comparative transcriptome analysis reveals molecular strategies of oriental river prawn *Macrobrachium nipponense* in response to acute and chronic nitrite stress. Fish Shellfish Immunol. 48, 254–265. 10.1016/j.fsi.2015.12.005 26687531

[B52] Yang Y. Yu H. Li H. Wang A. (2016). Transcriptome profiling of grass carp (*Ctenopharyngodon idellus*) infected with *Aeromonas hydrophila*. Fish Shellfish Immunol. 51, 329–336. 10.1016/j.fsi.2016.02.035 26945937

[B53] Yu W.-C. He C. Wu C. -L. Wang J. Li Z. Guo T. (2015). Extraction and identification of melanin in the shell and mantle of Pacific oyster *Crassostrea gigas*. Oceanol. Limnol. Sin. 46, 909–914. 10.11693/hyhz20150300065

[B54] Zhang G. Fang X. Guo X. Li L. Luo R. Xu F.. (2012). The oyster genome reveals stress adaptation and complexity of shell formation. Nature 490, 49–54. 10.1038/nature11413 22992520

[B55] Zheng H. Liu H. Zhang T. Wang S. Sun Z. Liu W. (2010). Total carotenoid differences in scallop tissues of *Chlamys nobilis* (Bivalve: Pectinidae) with regard to gender and shell colour. Food Chem. 122, 1164–1167. 10.1016/j.foodchem.2010.03.109

[B56] Zheng H. Zhang Q. Liu H. Liu W. Sun Z. Li S. (2012). Cloning and expression of vitellogenin (Vg) gene and its correlations with total carotenoids content and total antioxidant capacity in noble scallop *Chlamys nobilis* (Bivalve: Pectinidae). Aquaculture 366–367, 46–53. 10.1016/j.aquaculture.2012.08.031

