# Supplementary material for: A Preliminary Study on the Pattern, the Physiological Bases and the Molecular Mechanism of the Adductor Muscle Scar Pigmentation in Pacific Oyster Crassostrea gigas
Source: Front Physiol. 2017 Sep 12;8:699. doi: 10.3389/fphys.2017.00699 (PMC5600958; doi:10.3389/fphys.2017.00699)
Supplement: Supplementary file 1 [file Table1.DOCX]

**Table S1.** The detailed data of the pigmentation patterns of adductor muscle scar and outer surface of shell

|  |  | **Number** |  | **Number** |  | **Number** |  | **Number** |  | **Number** |  | **Number** | **Number of different individuals** | **Percentage of different individuals** |
| --- | --- | --- | --- | --- | --- | --- | --- | --- | --- | --- | --- | --- | --- | --- |
| **Yantai group** | Complete white left shell | 59 | Complete white left muscle scar | 11 | Partial pigmented left shell | 215 | Complete white left muscle scar | 41 | Complete pigmented left shell | 26 | Complete white left muscle scar | 5 | 137 | 0.456666667 |
|  |  |  | Partial pigmented left muscle scar | 43 |  |  | Partial pigmented left muscle scar | 146 |  |  | Partial pigmented left muscle scar | 15 |  |  |
|  |  |  | Complete pigmented left muscle scar | 5 |  |  | Complete pigmented left muscle scar | 28 |  |  | Complete pigmented left muscle scar | 6 |  |  |
|  | Complete white right shell | 47 | Complete white right muscle scar | 16 | Partial pigmented right shell | 243 | Complete white right muscle scar | 54 | Complete pigmented right shell | 10 | Complete white right muscle scar | 1 | 137 | 0.456666667 |
|  |  |  | Partial pigmented right muscle scar | 27 |  |  | Partial pigmented right muscle scar | 151 |  |  | Partial pigmented right muscle scar | 7 |  |  |
|  |  |  | Complete pigmented right muscle scar | 4 |  |  | Complete pigmented right muscle scar | 38 |  |  | Complete pigmented right muscle scar | 2 |  |  |
| **Rushan group** | Complete white left shell | 57 | Complete white left muscle scar | 30 | Partial pigmented left shell | 226 | Complete white left muscle scar | 90 | Complete pigmented left shell | 17 | Complete white left muscle scar | 5 | 153 | 0.51 |
|  |  |  | Partial pigmented left muscle scar | 21 |  |  | Partial pigmented left muscle scar | 115 |  |  | Partial pigmented left muscle scar | 10 |  |  |
|  |  |  | Complete pigmented left muscle scar | 6 |  |  | Complete pigmented left muscle scar | 21 |  |  | Complete pigmented left muscle scar | 2 |  |  |
|  | Complete white right shell | 37 | Complete white right muscle scar | 12 | Partial pigmented right shell | 256 | Complete white right muscle scar | 84 | Complete pigmented right shell | 7 | Complete white right muscle scar | 5 | 158 | 0.526666667 |
|  |  |  | Partial pigmented right muscle scar | 19 |  |  | Partial pigmented right muscle scar | 128 |  |  | Partial pigmented right muscle scar | 0 |  |  |
|  |  |  | Complete pigmented right muscle scar | 6 |  |  | Complete pigmented right muscle scar | 44 |  |  | Complete pigmented right muscle scar | 2 |  |  |
| **Penglai group** | Complete white left shell | 34 | Complete white left muscle scar | 17 | Partial pigmented left shell | 216 | Complete white left muscle scar | 59 | Complete pigmented left shell | 50 | Complete white left muscle scar | 13 | 154 | 0.513333333 |
|  |  |  | Partial pigmented left muscle scar | 11 |  |  | Partial pigmented left muscle scar | 116 |  |  | Partial pigmented left muscle scar | 24 |  |  |
|  |  |  | Complete pigmented left muscle scar | 6 |  |  | Complete pigmented left muscle scar | 41 |  |  | Complete pigmented left muscle scar | 13 |  |  |
|  | Complete white right shell | 32 | Complete white right muscle scar | 12 | Partial pigmented right shell | 247 | Complete white right muscle scar | 63 | Complete pigmented right shell | 21 | Complete white right muscle scar | 3 | 145 | 0.483333333 |
|  |  |  | Partial pigmented right muscle scar | 14 |  |  | Partial pigmented right muscle scar | 127 |  |  | Partial pigmented right muscle scar | 12 |  |  |
|  |  |  | Complete pigmented right muscle scar | 6 |  |  | Complete pigmented right muscle scar | 57 |  |  | Complete pigmented right muscle scar | 6 |  |  |

**Table S4. GO terms enriched based on the upregulated genes in ‘black muscle’**

| GO_ID | GO_Term | Adjusted P value |
| --- | --- | --- |
| GO:0003676 | nucleic acid binding | 3.41E-21 |
| GO:0034641 | cellular nitrogen compound metabolic process | 8.45E-16 |
| GO:0006396 | RNA processing | 1.30E-15 |
| GO:0090304 | nucleic acid metabolic process | 2.21E-15 |
| GO:0006139 | nucleobase, nucleoside, nucleotide and nucleic acid metabolic process | 3.57E-15 |
| GO:0006807 | nitrogen compound metabolic process | 1.07E-13 |
| GO:0016779 | nucleotidyltransferase activity | 4.01E-12 |
| GO:0034660 | ncRNA metabolic process | 6.88E-11 |
| GO:0008152 | metabolic process | 1.46E-10 |
| GO:0044237 | cellular metabolic process | 5.09E-10 |

**Table S5. GO terms enriched based on the upregulated genes in ‘black mantle’**

| GO_ID | GO_Term | Adjusted P value |
| --- | --- | --- |
| GO:0009987 | cellular process | 4.32E-05 |
| GO:0005622 | intracellular | 5.92E-05 |
| GO:0005623 | cell | 5.92E-05 |
| GO:0044464 | cell part | 5.92E-05 |
| GO:0005618 | cell wall | 0.001182 |
| GO:0009847 | spore germination | 0.001182 |
| GO:0044424 | intracellular part | 0.005241 |
| GO:0044237 | cellular metabolic process | 0.005241 |
| GO:0005575 | cellular_component | 0.005241 |
| GO:0008150 | biological_process | 0.009186 |

**Table S6.** **The location information of the upregulated tyrosinase genes (red) and their neighboring tyrosinase genes (black) in scaffolds**

|  | **Gene ID** | **Scaffold No.** | **Scaffold Length** | **Start** | **End** | **+/-** |
| --- | --- | --- | --- | --- | --- | --- |
| **Muscle** | CGI_10011916 | scaffold43702 | **305484** | 231268 | 234872 | - |
|  | CGI_10011911 | scaffold43702 | **305484** | 180692 | 193700 | - |
|  | CGI_10011912 | scaffold43702 | **305484** | 197338 | 200147 | - |
|  | CGI_10011913 | scaffold43702 | **305484** | 210121 | 213690 | - |
|  | CGI_10012743 | Scaffold1792 | **336274** | 46300 | 55453 | - |
| **Mantle** | CGI_10013418 | Scaffold1630 | **356498** | 53535 | 55352 | + |
|  | CGI_10017214 | Scaffold248 | **495890** | 37096 | 41278 | - |
|  | CGI_10021076 | Scaffold203 | **674944** | 507685 | 510037 | + |
|  | CGI_10021075 | scaffold203 | **674944** | 495398 | 496914 | + |

**Table S7.** Retinol metabolism pathway was enriched based on the upregulated genes in ‘black mantle’ or ‘black muscle’

|  | **MapID** | **MapTitle** | **Pvalue** | **x** | **y** | **n** | **N** | **EnrichDirect** | **GeneIDs** | **annotation** |
| --- | --- | --- | --- | --- | --- | --- | --- | --- | --- | --- |
| mantle | 830 | Retinol metabolism | 4.83E-05 | 7 | 65 | 125 | 8347 | Over | CGI_10011065  CGI_10026868  CGI_10026867  CGI_10022185  CGI_10018485  CGI_10021866  CGI_10021688 | Cytochrome P450 1A1  Retinal dehydrogenase 1  Retinal dehydrogenase 1  Cytochrome P450 3A29  Diacylglycerol O-acyltransferase 1  Omega-crystallin  Aldehyde dehydrogenase |
| muscle | 830 | Retinol metabolism | 0.007878458 | 6 | 65 | 225 | 8347 | Over | CGI_10016640  CGI_10011491  CGI_10017766 CGI_10028005  CGI_10009121 CGI_10005487 | Cytochrome P450 1A2  Cytochrome P450 1A2  Cytochrome P450 26A1  Cytochrome P450 3A24  17-beta-hydroxysteroid dehydrogenase 13  Retinoic acid receptor responder protein 3 |
