# Supplementary material for: A Preliminary Study on the Pattern, the Physiological Bases and the Molecular Mechanism of the Adductor Muscle Scar Pigmentation in Pacific Oyster Crassostrea gigas
Source: Front Physiol. 2017 Sep 12;8:699. doi: 10.3389/fphys.2017.00699 (PMC5600958; doi:10.3389/fphys.2017.00699)
Supplement: Supplementary file 4 [file Image1.PDF]

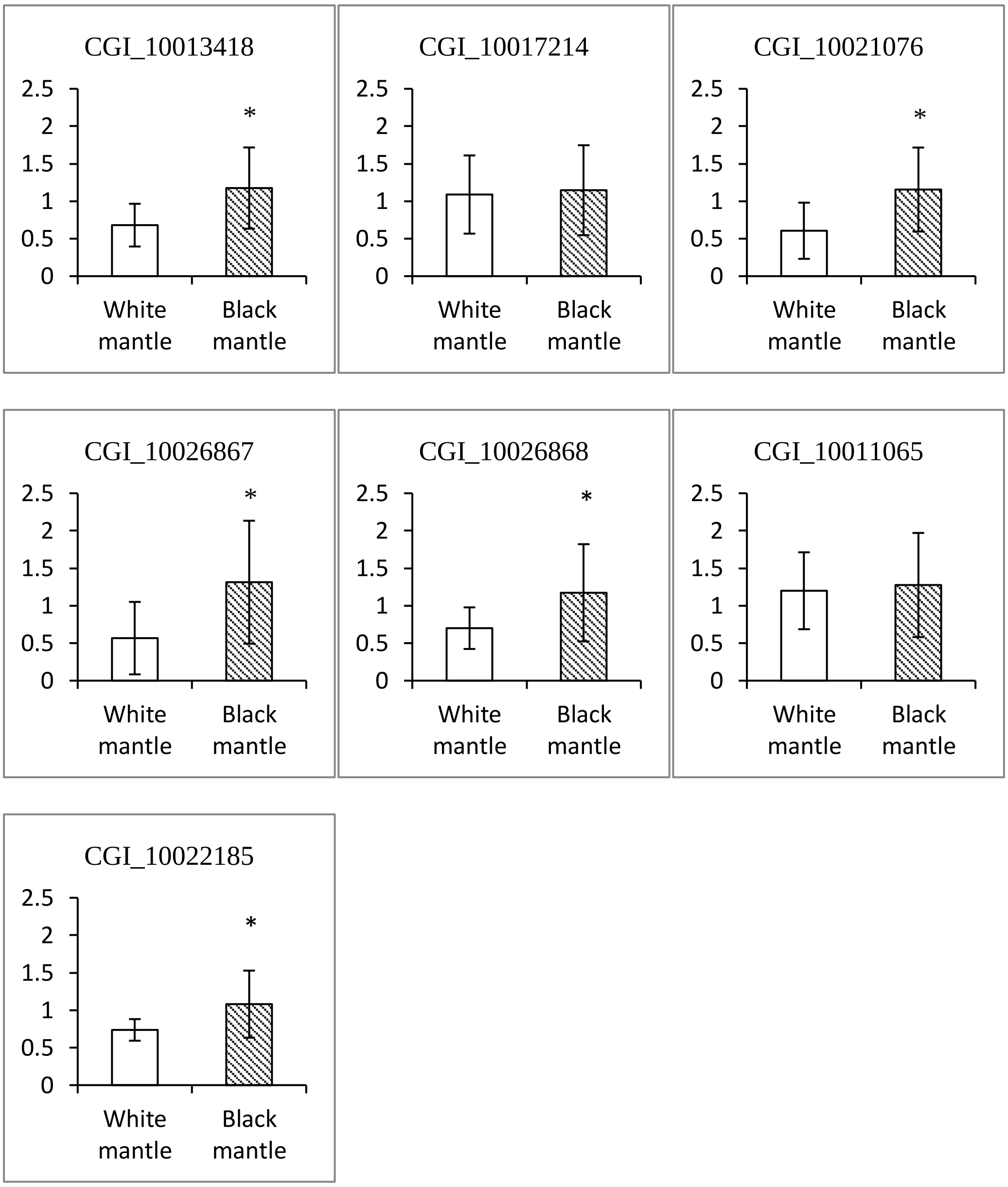

Figure S1. The expression levels of seven genes (Tyrosinase, Retinal dehydrogenase and Cytochrome P450 genes) in the black or white mantles by qRT-PCR. Ordinate is the expression level of the gene ($2^{-\Delta\Delta CT}$); the abscissa the color of mantle; error bars represent standard errors; each group included 12 individuals; * and ** indicates the significant difference at 0.05 and 0.01 levels, respectively.
